# Late-onset Leber’s hereditary optic neuropathy and antiandrogens for prostate cancer: is there a causative link?

**DOI:** 10.3389/fneur.2025.1616992

**Published:** 2025-08-13

**Authors:** Giulia Amore, Michele Carbonelli, Diego D’Angeli, Luigi Bonan, Marco Faustini-Fustini, Alessandra Maresca, Valerio Carelli, Chiara La Morgia

**Affiliations:** ^1^Department of Medical and Surgical Sciences (DIMEC), Alma Mater Studiorum - University of Bologna, Bologna, Italy; ^2^Ophthalmology Unit, IRCCS Azienda Ospedaliero-Universitaria di Bologna, Bologna, Italy; ^3^Department of Biomedical and Neuromotor Sciences (DIBINEM), Alma Mater Studiorum - University of Bologna, Bologna, Italy; ^4^IRCCS Istituto delle Scienze Neurologiche di Bologna, Bologna, Italy

**Keywords:** Leber’s hereditary optic neuropathy, hormones, mitochondrial disease, androgen deprivation therapy, estrogens

## Abstract

**Introduction:**

Leber’s hereditary optic neuropathy (LHON) is a maternally inherited condition due to mitochondrial DNA (mtDNA) mutations usually affecting young men within their thirties, while women seem protected by estrogens with a female-to-male ratio of 1:3. Late-onset cases (over 40 years of age) are usually associated to toxic exposure to tobacco smoke or drugs causing mitochondrial dysfunction.

**Results:**

We describe two cases of LHON remarkable for their late onset (> 60 years) in the absence of classic toxic factors. They were both affected by advanced prostate cancer and developed LHON after introduction of enzalutamide, an antagonist of androgens’ receptor, in association with leuprolide, a gonadotropin-releasing hormone (GnRH) analogue, used in the context of Androgen deprivation therapy (ADT). Both patients presented very low serum levels of gonadotropin, estrogens and androgens compatible with hormonotherapy. MtDNA copy number in our probands resulted significantly reduced (like other LHON affected cases), compared to age-matched LHON unaffected mutation carriers and controls.

**Discussion:**

The role of hormones in LHON pathogenesis remains still debated. Recent evidence suggests a protective effect of estrogens in increasing mitochondrial biogenesis (and mtDNA copy number), partially explaining the gender bias of the disease, while the role of androgens is yet to be fully understood. Considering the effect of the ADT on circulating hormonal levels and their reciprocal interactions, we hypothesize that in a context of already low estrogens levels due to GnRH analogue, the block of androgens receptors by Leuprolide further imbalance the estrogens to androgens ratio and eventually trigger the disease.

## Introduction

Leber’s hereditary optic neuropathy (LHON) is a maternally inherited disease characterized by a selective degeneration of retinal ganglion cells (RGCs) due to mitochondrial dysfunction (1).

Three mitochondrial DNA (mtDNA) point mutations affecting the complex I subunits genes, namely m.11778G > A/MT-ND4, m.3460G > A/MT-ND1 and m.14484 T > C/MT-ND6, account for up to 90% of cases. RGCs, whose axons form the optic nerve acquiring the myelinated sheath only after crossing the lamina cribrosa, are among the most metabolically demanding cells in the human body, relying on a high blood flow-to-tissue volume ratio and a highly efficient mitochondrial system to generate ATP through oxidative phosphorylation (OXPHOS) in order to convey the signal along their intraretinal unmyelinated nerve fibers (2). In LHON, the pathogenic mtDNA mutations selectively disrupt the activity of complex I in the OXPHOS pathway, causing downstream bioenergetic failure and excessive reactive oxygen species (ROS) production, leading ultimately to RGC degeneration and optic atrophy (3).

Clinically, LHON typically occurs in young men in their thirties and presents with a subacute, painless loss of central vision affecting both eyes simultaneously or sequentially over a short period (weeks or months). Late-onset cases (> 40 years) are less common and are often associated with significant exposure to environmental stressors or toxics, such as prolonged tobacco use, excessive alcohol consumption or drugs causing mitochondrial dysfunction (4).

Women are less affected than men with a reported female-to-male ratio of around 1:3 due to a possible protective role of estrogens (5), which stimulate mitochondrial biogenesis (6), a known compensatory and protective mechanism in LHON (7), so that targeting estrogens receptors has been proposed as a preventive strategy (8). Currently, Idebenone, a coenzyme Q10 analogue with antioxidant properties that allows the transfer of electrons directly to Complex III, bypassing the Complex I block, is the only approved treatment for LHON in Europe. It has been shown that Idebenone therapy leads to a significant improvement of visual function compared to natural history (9). Another promising therapeutic strategy for LHON includes gene therapy based on the allotopic expression of the wild-type ND4 subunit in the nucleus, which was evaluated in clinical trials only for patients carrying the m.11778G > A/MT-ND4 mutation but it is not yet approved. The allotopic expression approach consists in a recombinant adeno-associated-virus-(AAV) vector containing the wild-type sequence of the defective mtDNA-encoded gene recoded to be expressed in the nucleus, associated with a mitochondrial targeting sequence allowing the mitochondrial import of the protein translated from cytoplasmic ribosome. The AAV2 vector containing the transgene construct is delivered to the retina via intravitreal injection (10).

Although influenced by the specific mutation, overall visual prognosis in LHON remains poor, with most patients experiencing persistent and significant visual impairment.

Here, we report two cases of late-onset LHON following hormonal (androgen-suppressing) therapy for prostate cancer, specifically after the introduction of Enzalutamide, an antagonist of androgens’ receptor, in association with Leuprolide, a gonadotropin-releasing hormone (GnRH) analogue. These cases offer new insights into the role of hormonal factors in LHON susceptibility and disease conversion, shedding light on their potential impact in triggering LHON.

## Case series

### Patient 1

A 61-year-old non-smoker male with a positive family history for LHON due to m.3460G > A/MT-ND1 mutation (mother and two nieces affected) and a personal history of depressive disorder was diagnosed for IV-stage prostate adenocarcinoma with bone and lymph nodes metastases at 59 years of age. He was treated with chemotherapy (Taxotere and Docetaxel from April to August 2020) and hormonotherapy with Leuprolide 11.25 mg/mL every 3 months since April 2020 and Denosumab (a monoclonal antiboby to the receptor activator of nuclear factor-κB ligand) monthly since July 2020. Enzalutamide 160 mg/day was added in October 2021.

**Figure 1:**
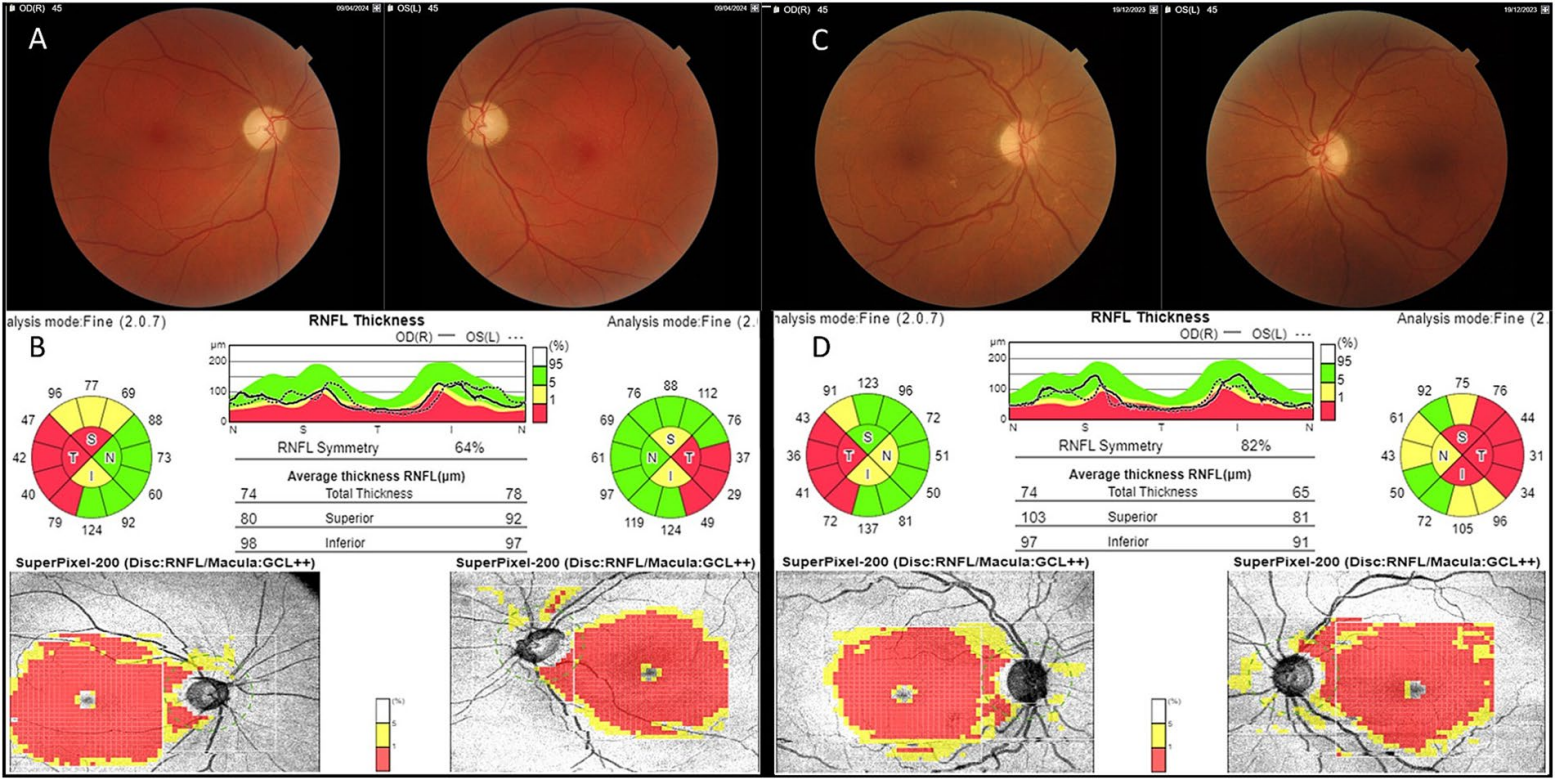
Fundus and OCT findings. RNFL, Retinal Nerve Fiber Layer; GCL, Ganglion Cell Layer. Patient 1: mild temporal optic pallor at fundus imaging **(A)** corresponding to bilateral RNFL thinning of the temporal quadrant with complete GCL defect at OCT **(B)** 8 months from LHON onset. Patient 2: mild temporal optic pallor at fundus imaging **(C)** corresponding to bilateral RNFL thinning of the temporal quadrant with complete GCL defect at OCT **(D)** 8 months from LHON onset.

He presented with acute painless central visual loss in left eye (OS) in August 2023, followed by right eye (OD) in September 2023. Fundoscopic examination revealed papillary hyperemia with mild swelling of the nerve fibers (the so called “pseudoedema” which is typically seen in the acute phase of LHON and often misdiagnosed with papillitis). Optical coherence tomography (OCT) confirmed increased thickness of the peripapillary retinal nerve fiber layer (RNFL) in inferior and temporal quadrants with initial perifoveal defect in Ganglion Cell Layer (GCL) OS>OD. Visual fields revealed bilateral centro-cecal scotoma, more evident in the left eye. All these elements pointed toward suspicion of LHON onset, which was rapidly confirmed by genetic testing due to the positive family history and the patient was started on Idebenone therapy at the dosage of 900 mg/day since October 2023. The patient reached the nadir with Best Corrected Visual Acuity (BCVA) of “counting fingers” bilaterally in April 2024 and started experiencing a small recovery of vision in OD (BCVA 0.02 decimal) after 14 months from onset (October 2024). Figures 1A,B shows typical temporal optic nerve atrophy at fundus photography and at OCT 8 months after onset.

A complete evaluation of pituitary-gonadal function was performed at time of LHON diagnosis as suggested by our endocrinologist (details on hormonal levels are reported in Table 1). As expected by ongoing suppressive hormonal therapy, all gonadotropins, estrogens and androgens levels resulted very low. Meanwhile, the patient presented progression of prostate cancer with increasing values of prostate specific antigen (PSA) since June 2024 from 24 ng/mL up to 93 ng/mL in October 2024 (normal value < 3.1 ng/mL). Therefore, the oncologist decided to withdraw Enzalutamide and added Abiratenone (an androgen biosynthesis inhibitor) 1,000 mg/day. In November 2024, due to local disease progression, chemotherapy with Cabazitaxel and palliative radiation therapy were also started.

**Table 1:** Hormonal status of patient 1 and 2 at time of LHON diagnosis.

Patient	LH (mUI/mL; nv 1.2–8.6)	FSH (mUI/mL; nv 1.3–19.3)	Oestradiol (pg/mL; nv < 166)	DHEA (μg/dL; nv 24–244)	Testosterone (ng/mL; nv 6.1–27)	Free testosterone (pg/mL; nv 32–168)	Δ4-Androstenediol (ng/mL; nv 0.4–2.6)
1	<0.2	2.9	15	128.6	0.00	NA	0.74
2	<0.2	3.8	14	81	0.42	1.28	1.18

LH, luteinizing hormone; FSH, follicle stimulating hormone; DHEA, dehydroepiandrosterone.

### Patient 2

A 77-year-old non-smoker male with a negative family history for LHON reported a personal medical history of prostatic adenocarcinoma since 65 years of age, for which he was initially subjected to radical prostatectomy and radiotherapy and subsequently treated with Leuprolide 11.25 mg/mL every 3 months for 11 years (since 2011). Enzalutamide 160 mg/day was added to hormonotherapy since September 2022.

He presented subacute and bilateral onset of painless visual loss in April 2023 with evidence of bilateral central scotoma at visual fields. Autoimmune screening was negative, including AQP4-IgG and MOG-IgG. Cerebrospinal fluid analysis was normal, with absence of oligoclonal bands. Brain MRI showed bilateral FLAIR-hyperintense lesion affecting the optic chiasm and the optic tracts up to lateral geniculate bodies without contrast enhancement. To rule out a paraneoplastic manifestation, serum dosage of anti-neuronal antibodies and neoplastic markers was performed resulting negative and a total body contrast-enhanced CT-scan did not reveal any other localization of neoplastic lesions, except for the prostate. He was initially treated with high-dose corticosteroids, without clinical benefit. We then performed genetic testing, disclosing the presence of the homoplasmic m.14484 T > C/MT-ND6 mutation pathogenic for LHON and the patient received Idebenone at the dosage of 900 mg/die since October 2023. Visual nadir was reached in September 2023, with BCVA of “counting finger” in OD and 0.01 decimal in OS, he then experienced some visual recovery (last BCVA 0.16 OD and 0.125 OS in March 2025).

Figures 1C,D shows fundus imaging and OCT results 8 months after onset. Hormonal status during hormonotherapy is presented in Table 1; as shown, both gonadotropin and sex hormone levels were consistently very low.

In December 2023 he presented progression of prostate cancer, as revealed by increase of PSA value (from 3.85 ng/mL in January 2024 to 34.7 ng/mL in July 2024) and evidence of lymph node involvement detected on the PET scan in January 2024. Oncologists decided to withdraw Enzalutamide and add Abiraterone 1,000 mg/day until July 2024; in September 2024, chemotherapy with Docetaxel was started after evidence of further disease progression.

### Assessment of mtDNA copy number in blood cells

**Figure 2:**
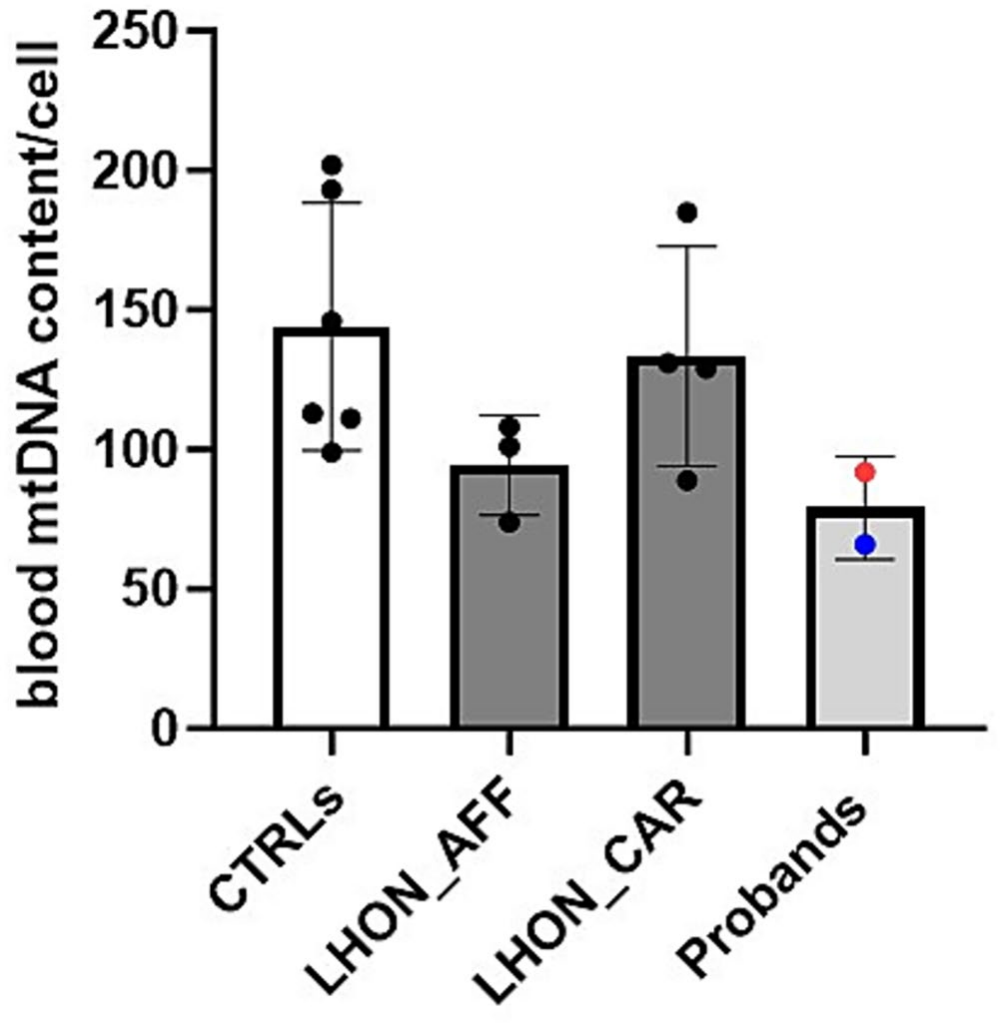
MODIFY WITH: MtDNA content assessed in blood cells from healthy controls (CTRLs), LHON affected patients (LHON_AFF), LHON unaffected carriers (LHON_CAR), Probands (Patient 1 blue dot, Patient 2 red dot). Probands showed mtDNA content comparable to other LHON affected patients, but reduced compared with the healthy controls and LHON carriers. However, no statistically significant difference was found.

DNA was isolated from the buffy coat using the Maxwell 16 automated instrument. Quantification of mtDNA copy number relative to nuclear DNA was performed by Real Time PCR with a multiplex probe-based method, as previously described (11). We assessed mtDNA copy number in age-matched healthy controls (CTRLs, *n* = 6), LHON affected patients (*n* = 3), LHON unaffected mutation carriers (*n* = 4) and our two probands (Patients 1 and 2). Demographic and genetic details of controls, LHON affected and carriers and of the two probands are reported in Table 2. The evaluation of mtDNA copy number has been performed at 23 and 10 months after the introduction of Enzalutamide, respectively, for Patient 1 and 2. The results are shown in Figure 2. Despite the lack of statistical significance, possibly due to the limited number of individuals investigated, we note that the two probands of this study presented a reduced amount of mtDNA copies, in the low range of the other LHON affected individuals, as compared with both controls and LHON unaffected mutation carriers.

**Table 2:** Demographic characteristics and genetic status of controls, LHON affected, LHON carriers and probands.

Subjects	Gender	Age (mean±dev.st)	m.3460G > A/MT-ND1	m.14484 T > C/MT-ND6	m.11778G > A/MT-ND4
CTRLs (*n* = 6)	Males	57 ± 5	-	-	-
LHON affected (*n* = 3)	Males	48 ± 1	1	1	1
LHON carrier (*n* = 4)	Males	48 ± 10	2	1	1
Probands (*n* = 2)	Males	69 ± 11	1	1	

## Discussion

In this study, we report the cases of two male patients carrying a mtDNA mutation pathogenetic for LHON, both diagnosed with prostate cancer and experiencing late-onset conversion following the introduction of an androgen receptor antagonist (Enzalutamide) to a longstanding hormonotherapy with a GnRH analogue (Leuprolide). Notably, neither patient had a history of tobacco smoking or excessive alcohol consumption. Although causality cannot be firmly established, the association with androgen-suppressive therapy and the absence of alternative triggers point to a possible causal link. Moreover, the late onset of the disease further reinforces the notion that external stressors play a relevant role in tipping the balance toward disease manifestation (4).

Given the central role of mitochondrial dysfunction in LHON, investigating the interplay between hormonal regulation and mitochondrial homeostasis could provide key insights into disease susceptibility. An intriguing feature of LHON is, in fact, its marked male predominance, with a male-to-female ratio of approximately 3:1 (5). The underlying reasons for this gender disparity remain not totally understood. One hypothesis is that differences in sex hormones between males and females may contribute to this imbalance with a protective role of estrogens in LHON driven by stimulation of mitochondrial biogenesis (6). Notably, RGCs express both androgen and estrogen receptors, and the retina possesses intrinsic steroidogenic activity, including the ability to convert testosterone through aromatization (12). The most compelling evidence for a hormonal influence in LHON implicates estrogens. A growing body of research suggests in fact that estrogens offer neuroprotection in various neuropathological conditions (13–17). *In vitro* studies demonstrate that estrogen exposure in LHON cybrids enhances mitochondrial biogenesis, optimizes bioenergetic efficiency, reduces oxidative stress, and ultimately improves cell viability by reducing apoptosis (6–8). Interestingly, Fantini and colleagues reported a case of LHON in a menopausal woman carrying the m.10197G > A/ND3 mtDNA mutation who developed vision loss shortly after discontinuing hormone replacement therapy (HRT). Her vision improved within a month and was fully restored by 8 months following treatment with Idebenone and HRT, a recovery trajectory that exceeds the expected effect of Idebenone alone, suggesting a potential therapeutic benefit from hormonal modulation (18). Consistently, large-scale epidemiological data indicate that the male-to-female conversion ratio approaches 1:1 during prepubertal and postmenopausal stages, when estrogen levels in females are at their nadir, strengthening estrogen’s protective role in disease conversion (5). Notably, although the difference was not statistically significant, our two patients exhibited a reduced mtDNA copy number in blood cells -similar to other LHON-affected patients- when compared with controls and LHON-unaffected mutation carriers. This finding is consistent with our previous study, which highlighted the protective role of increased mitochondrial biogenesis (7).

Androgen deprivation therapy (ADT) is a cornerstone of advanced prostate cancer treatment. Enzalutamide, a nonsteroidal agent, inhibits peripheral androgen signaling by binding to and blocking the androgen receptor (AR), while Leuprolide, a GnRH analogue, suppresses gonadal steroidogenesis by inhibiting gonadotropin release. Whether used alone or in combination, these agents result in profound testosterone activity suppression. In this context, given previous premises, the difficulty is to explain how low serum testosterone becomes a putative risk for developing late-onset LHON in our cases. In fact, serum testosterone levels in men as well as serum oestradiol levels in women had been by far considered the most prevalent differences in terms of gender for a long time. However, a growing body of evidence has shown that estrogen deficiency has a relevant role in the pathophysiology of a number of diseases also in men (19, 20). Both our patients were treated with Leuprolide, which inhibits gonadotropin release, as a result, serum testosterone dramatically decreased, reducing also the amount of testosterone available to be metabolized to oestradiol by aromatase activity in peripheral tissues. It is of note that in our patients serum oestradiol (10–15 ng/mL see Table 1) was reduced by half compared to that measured in normal men (21). In this clinical setting, the reduced serum oestradiol, considering the protective role of estrogens in LHON, may have played a role in favoring the development of the disease.

On the other hand, the potential role of androgens in LHON remains largely unexplored and speculative. LHON most commonly manifests in young men (ages 20–40), coinciding with peak testosterone levels. Mounting evidence suggests that androgens influence mitochondrial bioenergetics, oxidative stress regulation, and apoptosis, all of which are relevant to LHON pathophysiology [for a review, see (22)]. Jankauskaitė and others investigated the effects of testosterone on LHON cybrid (m.11778G > A) cells and found that exposure to testosterone increased apoptotic cell death, implying a potential role in modulating disease susceptibility (23). That said, preclinical data also support a protective role of testosterone in aging-related neuronal mitochondrial dysfunction (24). In this complex scenario it is interesting to point out that both patients experienced visual loss 7 to 22 months after the administration of Enzalutamide, implying a role of AR blocking as a potential precipitating factor in LHON together with the prolonged effect of Leuprolide in reducing estrogen levels. Recent evidence shows that Enzalutamide affects mitochondrial metabolism in AR-driven diseases through negative regulation of the transcription of the mitochondrial pyruvate carrier (MPC), which enhances tumor proliferation (25). *In vitro* studies also show that Enzalutamide increases levels of caspase-9 and proapoptotic Bax protein in glioblastoma cells, amplifying cell death mechanism and thus not excluding a possible toxic effect on mitochondrial function (26). It is well known that AR inhibitors may lead to initial response in advanced prostate cancer; however, the disease sometimes progress to castration-resistant prostatic cancer (CRPC), and both our patients represent a clear example of such a possibility. Interestingly, they developed late-onset LHON just before or nearly simultaneously the occurrence of biochemical evidence of relapse and progress of prostate cancer. Over the last decade, several studies have been designed in order to understand the mechanisms responsible for CRPC. In a recent elegant *in vitro* study, Blomme and collaborators showed that long-term-resistance to AR inhibitors (bicalutamide, apalutamide, and enzalutamide) was due to aberrant AR signaling and that 2.4-dienoyl-CoA reductase, an axillary mitochondrial enzyme involved in polyunsaturated fatty acid degradation and *β*-oxidation, could be a relevant biomarker for CRPC (27). However, the putative role of mitochondrial involvement in AR inhibitor resistance remains to be clarified. It is possible that individuals with a genetic predisposition as our patients exhibit a delicate balance between sexual hormones metabolism and mitochondrial function, being prone to develop RGCs damage as a consequence of a sudden modification of this homeostasis.

## Conclusion

These two cases of late onset LHON are remarkable for the possible role of androgen-suppressive therapy as a never reported before trigger of the disease. This could be relevant considering the large use of these drugs in the treatment of prostatic cancer, which is one of the most common neoplastic diseases in adult men. Moreover, our clinical observation support once again the hypothesis that hormonal factors may have a major impact in LHON pathogenesis, considering both the suggested protective effect of estrogens and mitochondrial biogenesis, and the not yet fully understood role of androgens. The evidence of reduced mtDNA copy number in our probands further supports our hypothesis. We hypothesize that in a context of already low estrogens levels due to GnRH analogue, the block of androgens receptors further imbalance the intracellular estrogens to androgens ratio and eventually trigger the disease. A possible toxic effect of Enzalutamide on mitochondrial function should be also considered in this context.

Further research is needed to elucidate the underlying mechanisms of this effect and its relevance to LHON pathophysiology.

## Data Availability

All relevant data is contained within the article: the original contributions presented in the study are included in the article, further inquiries can be directed to the corresponding author.

## Author’s note

Prof. Valerio Carelli and Dr. Chiara La Morgia are full members of the EURO-NMD.
